# Ingestion of Free Amino Acids Compared with an Equivalent Amount of Intact Protein Results in More Rapid Amino Acid Absorption and Greater Postprandial Plasma Amino Acid Availability Without Affecting Muscle Protein Synthesis Rates in Young Adults in a Double-Blind Randomized Trial

**DOI:** 10.1093/jn/nxab305

**Published:** 2021-10-12

**Authors:** Michelle E G Weijzen, Rob J J van Gassel, Imre W K Kouw, Jorn Trommelen, Stefan H M Gorissen, Janneau van Kranenburg, Joy P B Goessens, Marcel C G van de Poll, Lex B Verdijk, Luc J C van Loon

**Affiliations:** Department of Human Biology, School of Nutrition and Translational Research in Metabolism (NUTRIM), Maastricht University Medical Centre+, Maastricht, The Netherlands; Department of Intensive Care Medicine, School of Nutrition and Translational Research in Metabolism (NUTRIM), Maastricht University Medical Centre+, Maastricht, The Netherlands; Department of Surgery, School of Nutrition and Translational Research in Metabolism (NUTRIM), Maastricht University Medical Centre+, Maastricht, The Netherlands; Department of Human Biology, School of Nutrition and Translational Research in Metabolism (NUTRIM), Maastricht University Medical Centre+, Maastricht, The Netherlands; Department of Human Biology, School of Nutrition and Translational Research in Metabolism (NUTRIM), Maastricht University Medical Centre+, Maastricht, The Netherlands; Department of Human Biology, School of Nutrition and Translational Research in Metabolism (NUTRIM), Maastricht University Medical Centre+, Maastricht, The Netherlands; Department of Human Biology, School of Nutrition and Translational Research in Metabolism (NUTRIM), Maastricht University Medical Centre+, Maastricht, The Netherlands; Department of Human Biology, School of Nutrition and Translational Research in Metabolism (NUTRIM), Maastricht University Medical Centre+, Maastricht, The Netherlands; Department of Intensive Care Medicine, School of Nutrition and Translational Research in Metabolism (NUTRIM), Maastricht University Medical Centre+, Maastricht, The Netherlands; Department of Surgery, School of Nutrition and Translational Research in Metabolism (NUTRIM), Maastricht University Medical Centre+, Maastricht, The Netherlands; Department of Human Biology, School of Nutrition and Translational Research in Metabolism (NUTRIM), Maastricht University Medical Centre+, Maastricht, The Netherlands; Department of Human Biology, School of Nutrition and Translational Research in Metabolism (NUTRIM), Maastricht University Medical Centre+, Maastricht, The Netherlands

**Keywords:** digestion, stable isotopes, splanchnic extraction, skeletal muscle, nutrition

## Abstract

**Background:**

The rate of protein digestion and amino acid absorption determines the postprandial rise in circulating amino acids and modulates postprandial muscle protein synthesis rates.

**Objective:**

We sought to compare protein digestion, amino acid absorption kinetics, and the postprandial muscle protein synthetic response following ingestion of intact milk protein or an equivalent amount of free amino acids.

**Methods:**

Twenty-four healthy, young participants (mean ± SD age: 22 ± 3 y and BMI 23 ± 2 kg/m^2^; sex: 12 male and 12 female participants) received a primed continuous infusion of l-[ring-^2^H_5_]-phenylalanine and l-[ring-3,5–^2^H_2_]-tyrosine, after which they ingested either 30 g intrinsically l-[1–^13^C]-phenylalanine–labeled milk protein or an equivalent amount of free amino acids labeled with l-[1–^13^C]-phenylalanine. Blood samples and muscle biopsies were obtained to assess protein digestion and amino acid absorption kinetics (secondary outcome), whole-body protein net balance (secondary outcome), and mixed muscle protein synthesis rates (primary outcome) throughout the 6-h postprandial period.

**Results:**

Postprandial plasma amino acid concentrations increased after ingestion of intact milk protein and free amino acids (both *P* < 0.001), with a greater increase following ingestion of the free amino acids than following ingestion of intact milk protein (*P*-time × treatment < 0.001). Exogenous phenylalanine release into plasma, assessed over the 6-h postprandial period, was greater with free amino acid ingestion (76 ± 9%) than with milk protein treatment (59 ± 10%; *P* < 0.001). Ingestion of free amino acids and intact milk protein increased mixed muscle protein synthesis rates (*P*-time < 0.001), with no differences between treatments (from 0.037 ± 0.015%/h to 0.053 ± 0.014%/h and 0.039 ± 0.016%/h to 0.051 ± 0.010%/h, respectively; *P*-time × treatment = 0.629).

**Conclusions:**

Ingestion of a bolus of free amino acids leads to more rapid amino acid absorption and greater postprandial plasma amino acid availability than ingestion of an equivalent amount of intact milk protein. Ingestion of free amino acids may be preferred over ingestion of intact protein in conditions where protein digestion and amino acid absorption are compromised.

See corresponding editorial on page 3.

## Introduction

Protein ingestion is an important anabolic stimulus that regulates muscle mass maintenance (1–3). The increase in muscle protein synthesis rates following protein ingestion has been attributed to the postprandial rise in circulating essential amino acids (EAAs), with the rise in plasma leucine concentration being of particular relevance (4). As the postprandial rise in circulating amino acids represents the driver of the muscle protein synthetic response to feeding, it is evident that protein digestion and amino acid absorption kinetics form a key determinant of the muscle protein synthetic response to protein intake (5–8). In agreement with this observation, the greater postprandial increase in circulating amino acids after the ingestion of whey compared with micellar casein has been proposed to be, at least partly, responsible for the greater anabolic properties of whey than casein (5, 6, 9, 10). As such, more rapidly digestible proteins (like whey) are considered more potent in stimulating muscle protein synthesis than more slowly digestible proteins (like micellar casein).

Early work in pigs has shown that following protein digestion and absorption ∼50% of dietary protein–derived amino acids are extracted by the splanchnic tissues in order to maintain functional mass (11). In agreement with these findings, studies in humans have demonstrated that 50–60% of dietary protein–derived leucine and phenylalanine is released in the circulation throughout a 5- to 6-h postprandial period, with the remainder likely retained in the splanchnic area (3, 12, 13). Interestingly, the percentage of the dietary protein–derived amino acids extracted by the splanchnic tissues seems to depend on various factors, including protein digestion and amino acid absorption kinetics (14). In support of these findings, we have previously shown that compared with the ingestion of its intact protein, ingestion of a protein hydrolysate leads to greater release of protein-derived phenylalanine into the circulation, which tends to further increase postprandial muscle protein synthesis rates (8). As exhibited intestinal absorption of free amino acids (FAAs) is more rapid than that of dietary protein–derived amino acids (15), it has been speculated that following ingestion of FAAs the amount of postprandial splanchnic amino acid retention is lower than that following the ingestion of an equivalent amount of intact protein. Consequently, ingestion of FAAs may result in a greater postprandial release of amino acids in the circulation, thereby further increasing postprandial muscle protein synthesis rates compared with the ingestion of an equivalent amount of intact protein.

In this study, protein digestion and amino acid absorption kinetics and the subsequent muscle protein synthetic response were compared after ingestion of intact milk protein (PRO) and following ingestion of an equivalent amount of crystalline FAAs. We hypothesized that the ingestion of FAAs would result in more rapid amino acid absorption, greater postprandial exogenous amino acid release into the circulation, and a greater increase in mixed muscle protein synthesis rates when compared with the ingestion of an equivalent amount of intact PRO in vivo in humans. To test this hypothesis, healthy, young individuals received a continuous infusion with l-[ring-^2^H_5_]-phenylalanine while consuming either a single bolus of specifically produced intrinsically l-[1-^13^C]-phenylalanine–labeled PRO (16–18) or an equivalent amount of FAAs enriched with free l-[1-^13^C]-phenylalanine. Combining the ingestion of free or (milk) protein bound l-[1-^13^C]-phenylalanine with continuous intravenous infusion of l-[ring-^2^H_5_]-phenylalanine allows us to assess protein digestion and amino acid absorption kinetics, quantify the amount of exogenous amino acids being released into the circulation, assess the postprandial rise in muscle protein synthesis rates, and directly assess the metabolic fate of the ingested free or protein-bound l-[1-^13^C]-phenylalanine (16). To our knowledge, this is the first study to provide a comprehensive overview of the differences in postprandial protein digestion and amino acid absorption kinetics and postprandial muscle protein synthesis rates following the ingestion of a single bolus of dietary protein compared with an equivalent amount of free crystalline amino acids in vivo in humans.

## Methods

### Study design

Twenty-four healthy, young participants (22 ± 3 y; 23 ± 2 kg/m^2^; 12 male/12 female) were recruited via advertisements on poster boards and social media to participate in a stable isotope infusion trial. This double-blind, randomized, parallel-group trial was conducted between February and August 2018 at Maastricht University Medical Centre+. A flowchart of subject enrollment is shown in **Supplemental Figure 1**. Participant characteristics are presented in **Table 1**. Participants were informed about possible risks of the experimental procedures prior to providing written informed consent. This study was approved by the local Medical Ethical Committee of Maastricht University Medical Centre+ and conforms to the principles outlined in the latest version of the Declaration of Helsinki for use of human subjects and tissue. This trial was registered at www.trialregister.nl as NTR6941. The study was independently monitored by the Clinical Trial Center Maastricht.

**TABLE 1:** Participant characteristics^1^

	PRO (*n =* 12)	FAA (*n =* 12)
Age, y	22 ± 2	23 ± 3
Sex: M/F, *n*	6/6	6/6
Weight, kg	70.0 ± 8.6	68.1 ± 10.6
BMI, kg/m^2^	22.8 ± 1.1	22.5 ± 2.9
Body fat, %	25.3 ± 6.6	24.2 ± 7.1
Appendicular lean mass, kg	22.9 ± 5.5	22.5 ± 5.7
Lean body mass, kg	50.9 ± 10.0	49.9 ± 10.1

^1^Values are means ± SDs. Data were analyzed with Student *t*-tests. No differences were detected between groups. FAA, free amino acid; PRO, milk protein.

### Pretesting

All participants were screened to assess body weight, height, body composition, and blood pressure. Body composition was determined by a DXA scan (Hologic Inc., Discovery A, QDR series, software package: APEX version 4.0.2). Whole-body and regional lean mass and fat mass were determined using reference values from the NHANES population-based dataset (19).

### Intact PRO and FAA mixture

Intrinsically l-[1-^13^C]-phenylalanine–labeled PRO was obtained by infusing lactating Holstein cows with l-[1-^13^C]-phenylalanine for 48 h while collecting milk, as described previously (16, 17, 20). A mixture of FAAs (Ajinomoto) was produced with an identical amino acid composition as the PRO and enriched with free l-[1-^13^C]-phenylalanine (Cambridge Isotope Laboratories) to achieve the same enrichment level. The l-[1-^13^C]-phenylalanine enrichment in both drinks was 38 mol % excess (MPE). The PRO and FAA mixture met all chemical and bacteriological specifications for human consumption. Participants consumed 30 g intrinsically l-[1-^13^C]-phenylalanine–labeled PRO or an equivalent amount of FAA, dissolved in 300 mL water (**Table 2**). Random assignment was performed by using a computerized random-number generator and stratified by sex. An independent person was responsible for random assignment and drink preparation.

**TABLE 2:** Amino acid contents of PRO and FAA treatments^1^

	% TAAs	
	PRO	FAA
EAAs		
Histidine	2.7	2.6
Isoleucine	4.9	4.8
Leucine	9.1	9.1
Lysine	7.7	9.5
Methionine	2.7	2.6
Phenylalanine	4.4	4.4
Threonine	4.2	4.2
Tryptophan	1.2	1.2
Valine	6.4	6.4
Total EAAs	43.2	44.7
NEAAs		
Alanine	3.0	2.9
Arginine	4.0	3.9
Aspartic acid^2^	7.3	7.2
Cysteine	0.8	0.8
Glutamic acid^3^	19.8	19.6
Glycine	1.8	1.8
Proline	9.9	9.6
Serine	5.3	5.3
Tyrosine	4.9	4.3
Total NEAAs	56.7	55.3

1 EAA, essential amino acids; FAA, free amino acids; NEAA, nonessential amino acids; PRO, milk protein; TAA, total amino acids.

2 Aspartic acid includes asparagine.

3 Glutamic acid includes glutamine.

### Diet and activity before testing

All participants were instructed to refrain from exhaustive physical activity and/or exercise and to maintain their usual dietary habits 3 d prior to the test day. All participants consumed a standardized meal [2.9 MJ; composed of 18% energy (En%) protein, 54 En% carbohydrate, and 28 En% fat] the evening prior to the experiment. Female subjects were tested in the first 10 d (follicular phase) of their menstrual cycle (standardized). There was an equal balance between males and females in each condition (6 males/females per condition).

### Experimental protocol

At 07:30 following an overnight fast, participants arrived at the laboratory by car or public transport. A Teflon cannula was inserted into an antecubital vein for isotope infusion and a second cannula was inserted into the dorsal hand vein of the contralateral arm for arterialized blood sampling (21). After a basal blood sample was collected (*t* = –210 min), the plasma phenylalanine and tyrosine pools were primed with a single intravenous dose of l-[ring-^2^H_5_]-phenylalanine (2 μmol/kg) and l-[ring-3,5-^2^H_2_]-tyrosine (0.613 μmol·kg^–1^). Subsequently, the continuous infusion was started (infusion rate: 0.05 μmol·kg^–1^·min^–1^ l-[ring-^2^H_5_]-phenylalanine and 0.015 μmol·kg^–1^·min^–1^ l-[ring-3,5-^2^H_2_]-tyrosine) and maintained for 9.5 h. Arterialized blood samples were collected during infusion at *t* = –120, –60, –30, 0, 15, 30, 45, 60, 90, 120, 180, 240, 300, and 360 min relative to drink ingestion for the analysis of plasma amino acid concentrations and enrichments and insulin concentrations. Blood samples (10 m l) were collected in EDTA-containing tubes and centrifuged at 1000 × *g* for 10 min at 4°C. Skeletal muscle biopsies were collected at *t* = –120 and 0 min for the determination of basal muscle protein synthesis rates. Immediately following the second biopsy (*t* = 0 min, from the contralateral leg), participants ingested a single bolus of 30 g intact PRO or an equivalent amount of FAAs. Additional muscle biopsies were collected at *t* = 120 and 360 min to determine postprandial muscle protein synthesis rates. Muscle biopsy collection was randomized between legs, and biopsies were collected from the middle region of the *M. vastus lateralis* (15 cm above the patella) using the Bergström needle technique (22). All biopsy samples were freed from any visible adipose tissue and blood. Aliquots of plasma and muscle samples were immediately frozen in liquid nitrogen and stored at –80°C until subsequent analysis.

### Plasma and muscle tissue analysis

The **Supplemental Methods** section presents details of analyses related to the determination of plasma (glucose; insulin; amino acids; l-[1-^13^C]- and l-[ring-^2^H_5_]-phenylalanine enrichments; and l-[1-^13^C]-, l-[ring-3,5-^2^H_2_]-, and l-[ring-^2^H_4_]-tyrosine enrichments) as well as muscle-derived (mixed muscle protein l-[1-^13^C]- and l-[ring-^2^H_5_]-phenylalanine enrichments) data.

### Western blotting

The Supplemental Methods section presents details of analysis related to the Western blotting performed on muscle tissue samples (at *t* = 0, 2, and 6 h).

## Calculations

Intravenous infusion of l-[ring-^2^H_5_]-phenylalanine and l-[ring-3,5-^2^H_2_]-tyrosine combined with the ingestion of free or (milk) protein–bound l-[1-^13^C]-phenylalanine, arterialized blood sampling, and skeletal muscle biopsy collection allowed us to assess postprandial protein handling (23). Specifically, metabolic calculations based on tracer kinetics were applied to assess whole-body amino acid kinetics (phenylalanine rate of appearance [R_a_], exogenous phenylalanine R_a_, endogenous phenylalanine R_a_, phenylalanine rate of disappearance [R_d_], and the fraction of dietary protein–derived phenylalanine release into the circulation), whole-body protein metabolism (whole-body protein synthesis, breakdown, and oxidation rate as well as whole-body net protein balance), and mixed muscle protein synthesis (muscle protein fractional synthesis rate [FSR] and incorporation of exogenous protein–derived phenylalanine in muscle protein) in the basal and/or postprandial state have been described in detail previously (23–25). Mixed muscle protein FSRs were calculated using the weighted mean plasma l-[ring-^2^H_5_]-phenylalanine enrichment during the incorporation period.

### Statistics

All data are expressed as means ± SDs. Normality of the data was verified using visual inspection of quantile–quantile plots and Shapiro-Wilk tests. No major violations for specific 2-way ANOVA assumptions were observed; in case of nonsphericity, the Greenhouse–Geisser correction was used. Differences in baseline characteristics were determined using unpaired, 2-tailed Student *t*-tests. Peak values and time to peak were calculated for all plasma time curves and unpaired, 2-tailed Student *t*-tests were applied to identify differences in peak values or time to peak between groups. Two-way ANOVA with time as the within-group factor and treatment as the between-group factor was used to compare differences between groups over time in plasma insulin, amino acid concentrations and enrichments, whole-body phenylalanine appearance rates, and FSR. In case of a significant interaction between time and treatment, separate analyses were performed to determine time effects for each group (1-factor repeated measures ANOVA) with a Bonferroni post hoc test to locate these differences and between-group effects for each time-point (Student *t*-test). Based upon previous studies (5, 8, 26), the expected difference in MPS between interventions would be 0.009%/h with an SD of 0.0065 (or ∼20% when expressed as relative difference between interventions). A sample size of 12 participants/group, including a 10% dropout rate. was calculated using a 2-sided statistical test (*P* < 0.05, 80% power), to detect differences in FSRs between groups (primary outcome). The effect size that could be detected was 1.38. Statistical significance was set at *P* < 0.05. All calculations were performed using the statistical software program SPSS (version 26.0, IBM Corp.).

## Results

### Plasma insulin and amino acid concentrations

**FIGURE 1:**
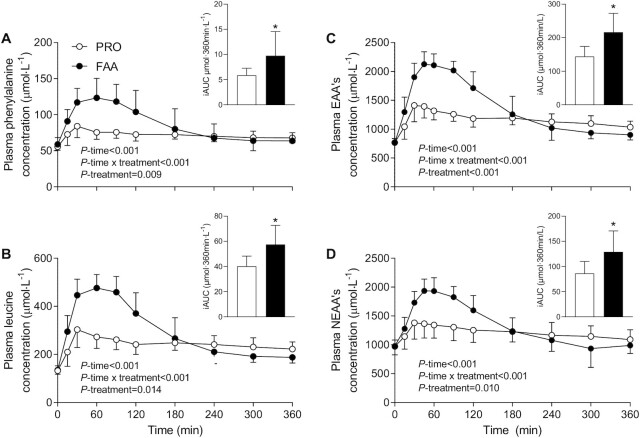
Plasma phenylalanine (A), leucine (B), EAA (C), and NEAA (D) concentrations and iAUC after ingesting 30 g PRO or an equivalent amount of FAAs. Values are means ± SDs, *n* = 12. Plasma concentrations were analyzed with the use of repeated measures ANOVA, iAUCs were analyzed with independent Student *t*-tests; *different from PRO (A: *P* = 0.043; B: *P* = 0.003; C: *P* = 0.006; D: *P* < 0.001). EAA, essential amino acid; FAA, free amino acid; iAUC, incremental AUC; NEAA, nonessential amino acid; PRO, milk protein.

Plasma insulin concentrations increased after the ingestion of the 30-g bolus of PRO or the equivalent amount of FAAs (*P*-time < 0.001), with a greater rise in plasma insulin concentrations following the ingestion of FAA compared with PRO (*P*-treatment = 0.014; *P*-time × treatment = 0.001; data not shown). Peak plasma insulin concentrations averaged 35 ± 15 and 24 ± 9 mU·L^–1^ in the FAA and PRO groups, respectively (*P* = 0.044). Plasma phenylalanine and leucine concentrations increased rapidly after the ingestion of PRO or the equivalent amount of FAA (*P*-time < 0.001; **Figure 1**). Plasma phenylalanine (*P*-treatment = 0.009; *P*-time × treatment < 0.001) and leucine (*P*-treatment < 0.001; *P*-time × treatment < 0.001) concentrations increased to a greater extent following FAA compared with PRO ingestion. Peak plasma phenylalanine and leucine concentrations were higher in the FAA compared with the PRO treatment (129 ± 7 compared with 87 ± 4 and 501 ± 42 compared with 326 ± 59 μmol·L^–1^, respectively; *P* < 0.001). In line with these results, the incremental AUCs (iAUCs) of plasma phenylalanine and leucine concentrations were greater with FAA than with PRO treatment (9.8 ± 4.8 compared with 5.9 ± 1.4 μmol·360 min·L^–1^ and 57.5 ± 15.2 compared with 40.1 ± 8.1 μmol·360min·L^–1^, respectively; *P* < 0.05). Postprandial plasma essential (Figure 1C) and nonessential amino acid (Figure 1D) concentrations increased following FAA and PRO ingestion (*P*-time < 0.001; *P*-treatment ≤ 0.010) and increased to a greater extent following FAA compared with intact PRO ingestion (*P*-time × treatment < 0.001).

### Plasma amino acid enrichments

**FIGURE 2:**
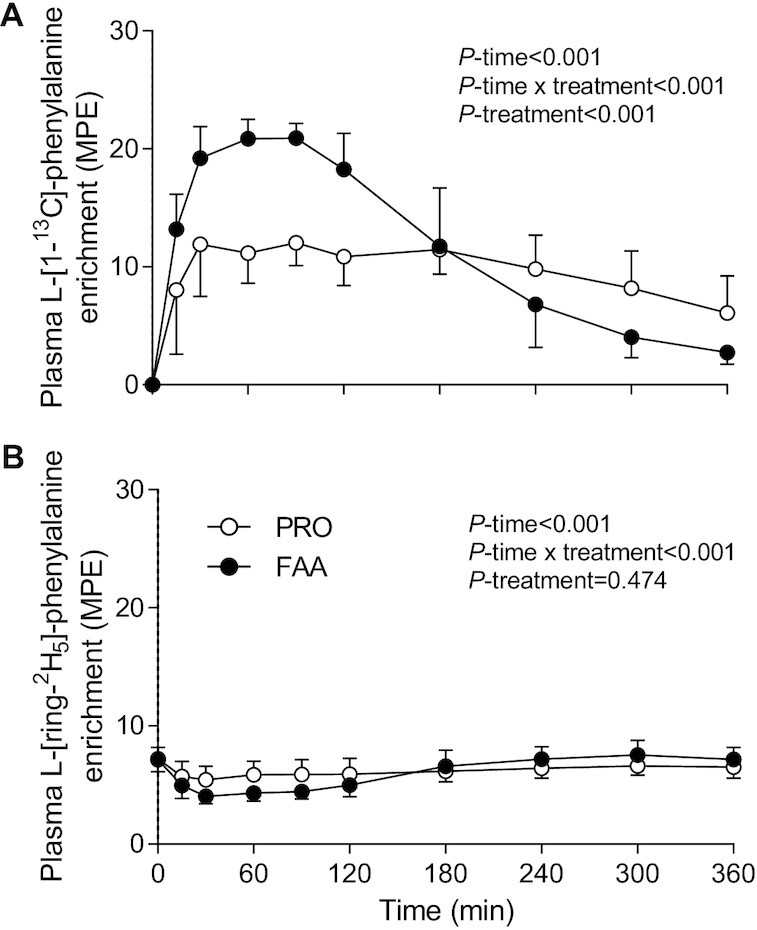
Plasma l-[1–^13^C]-phenylalanine (A) and l-[ring-^2^H_5_]-phenylalanine (B) after ingesting 30 g PRO or an equivalent amount of FAA. Values are means ± SDs, *n* = 12. Data were analyzed with the use of repeated measures ANOVA. FAA, free amino acid; MPE, mole % excess; PRO, milk protein.

Plasma l-[1-^13^C]-phenylalanine (tracer ingested as free l-[1-^13^C]-phenylalanine or as intrinsically l-[1-^13^C]-phenylalanine–labeled PRO) enrichments increased following FAA or PRO ingestion (*P-*time < 0.001; **Figure 2**A). Peak plasma l-[1-^13^C]-phenylalanine enrichments were reached at *t* = 90 min in both the FAA and PRO treatment groups. The increase in plasma l-[1-^13^C]-phenylalanine enrichments differed between treatments (*P*-time × treatment < 0.001), with higher values following FAA than with intact PRO ingestion until *t* = 180 min (*P* < 0.05). In contrast, plasma l-[1-^13^C]-phenylalanine enrichments were higher in the PRO compared with the FAA treatment following *t* = 240 min (*P* < 0.05). Plasma l-[ring-^2^H_5_]-phenylalanine (infused tracer) enrichments did not differ between treatments prior to FAA and PRO ingestion (*t* = 0 min; *P* > 0.05; Figure 2B). Plasma l-[ring-^2^H_5_]-phenylalanine enrichments were higher following intact PRO compared with FAA ingestion between *t* = 30 until *t* = 90 min (*P* < 0.05). Plasma l-[ring-^2^H_5_]-phenylalanine enrichments were higher in FAA compared with PRO at *t* = 300 min (*P* = 0.040).

### Whole-body amino acid kinetics

**FIGURE 3:**
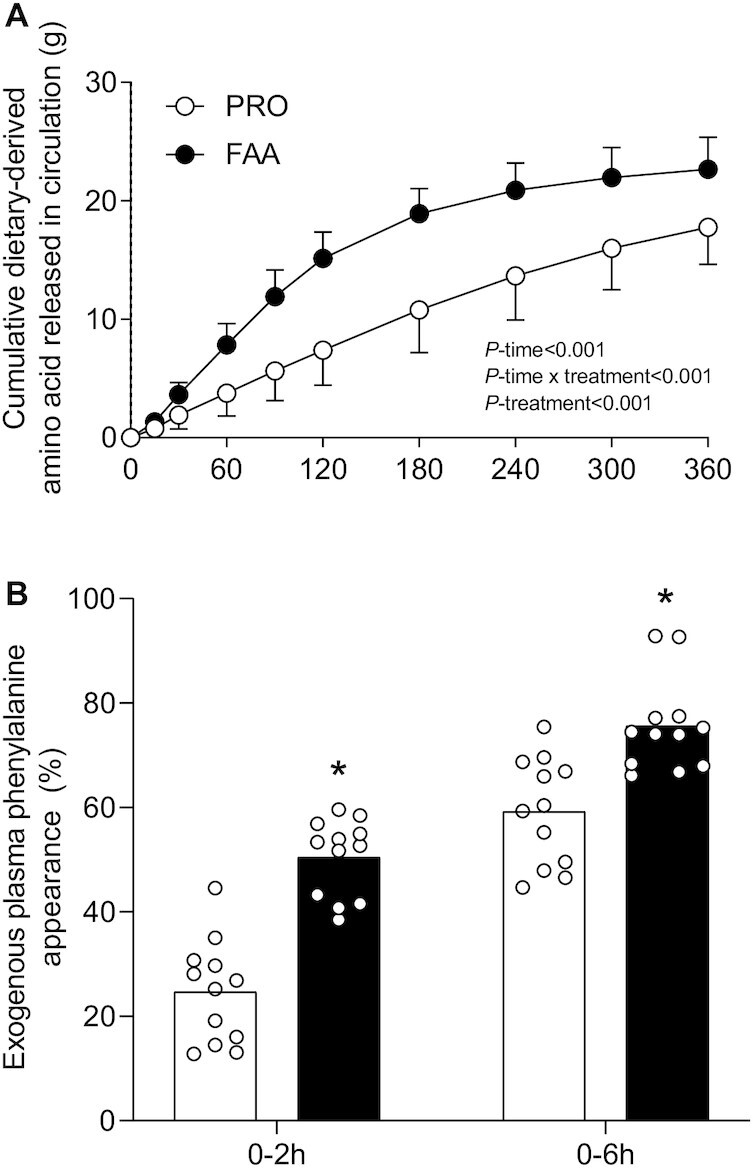
Cumulative dietary-derived amino acid released into the circulation (A), and postprandial exogenous phenylalanine availability (B), after ingesting 30 g PRO or an equivalent amount of FAAs. Bars are means (*n* = 12) and dots represent individual values. Data were analyzed with (A) repeated measures ANOVA and (B) independent Student *t*-tests. *Significantly different from PRO (*P* < 0.001). FAA, free amino acid; PRO, milk protein.

Cumulative dietary-derived amino acids released in the circulation (**Figure 3**A) increased following both FAA and PRO ingestion (*P*-time < 0.001), with a greater increase following FAA than PRO treatment (*P*-time × treatment < 0.001). Exogenous plasma phenylalanine availability (Figure 3B), calculated as the percentage of the total amount of phenylalanine consumed as FAAs or PRO that was released in the circulation during the 6-h postprandial period, was significantly higher following FAA than with PRO ingestion (76 ± 9% compared with 59 ± 10%; *P* < 0.001).

**FIGURE 4:**
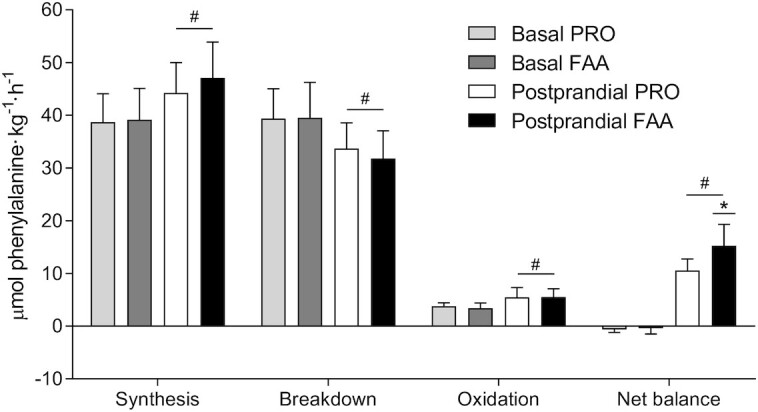
Whole-body protein synthesis, breakdown, oxidation, and net protein balance under basal conditions and after ingesting 30 g PRO or an equivalent amount of FAAs. Values are means ± SDs, *n* = 12. Data were analyzed with repeated measures ANOVA. *Significantly different from PRO (*P* = 0.002); ^#^Significantly different from basal (*P* < 0.001). FAA, free amino acid; PRO, milk protein.

In line with these findings, exogenous phenylalanine R_a_ (**Supplemental Figure 2A**) increased following FAA and PRO ingestion (*P*-time < 0.001), with peak levels being reached at *t* = 30 min in both treatments (0.75 ± 0.11 compared with 0.38 ± 0.13 μmol Phe·kg^–1^·min^–1^, respectively). Exogenous phenylalanine R_a_ increased to a greater extent in the FAA compared with the PRO treatment (*P*-time × treatment < 0.001). Endogenous phenylalanine R_a_ (Supplemental Figure 2B) decreased following FAA and PRO ingestion (*P*-time < 0.001), with a greater decline at *t* = 60–120 min following FAA than PRO ingestion (*P*-time × treatment = 0.004). FAAs and intact PRO ingestion both increased total phenylalanine appearance and disappearance rates (*P*-time < 0.001), with higher rates observed following FAA than with intact PRO ingestion (*P*-time × treatment < 0.001; Supplemental Figure 2C and D). Whole-body protein turnover rates are presented in **Figure 4**. Basal whole-body protein turnover rates did not differ between treatments (all *P* > 0.05). During the 6-h postprandial period, whole-body protein synthesis rates increased, protein breakdown rates decreased, and oxidation rates increased to a similar extent in both treatments (*P*-time < 0.001; *P*-treatment > 0.05; *P*-time × treatment > 0.05). Whole-body net protein balance increased from the basal to the postprandial state in both treatments (*P*-time < 0.001), with a greater increase following the ingestion of FAA than with PRO (15.3 ± 4.1 compared with 10.6 ± 2.2 μmol Phe·kg^–1^·h^–1^, respectively; *P*-treatment = 0.002; *P*-time × treatment = 0.002).

### Muscle protein synthesis and muscle protein–bound enrichments

**FIGURE 5:**
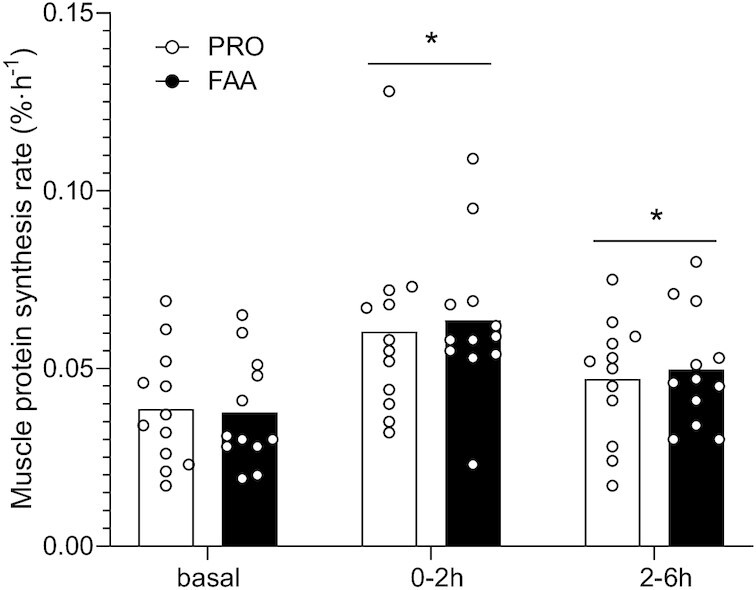
Mixed muscle protein fractional synthetic rates calculated based on l‐[ring‐^2^H_5_]‐phenylalanine tracer during the basal state and over the 6-h postprandial period after ingesting 30 g PRO or an equivalent amount of FAAs. Bars are means (*n* = 12) and dots represent individual values. *Significantly different from basal (*P* < 0.05). FAA, free amino acid; PRO, milk protein.

Basal mixed muscle protein FSR (based on l-[ring-^2^H_5_]-phenylalanine) averaged 0.037 ± 0.015 and 0.039 ± 0.016%·h^–1^ in the FAA and PRO treatment, respectively, with no differences between treatments (*P* = 0.868; **Figure 5**). Following ingestion of FAA or intact PRO, mixed muscle protein synthesis rates increased to 0.063 ± 0.021 compared with 0.060 ± 0.026%·h^–1^ (between *t* = 0–2 h) and 0.050 ± 0.016 compared with 0.047 ± 0.017%·h^–1^ (between *t* = 2–6 h) in the FAA and PRO treatment groups, respectively (*P*-time = 0.002), with no differences between treatments (*P*-time × treatment = 0.827). Mixed muscle protein synthesis rates assessed over the entire 6-h postprandial period (0.053 ± 0.014 compared with 0.051 ± 0.010%·h^–1^ following FAA compared with PRO ingestion, respectively) were significantly higher than basal mixed muscle protein synthesis rates (*P* < 0.001), with no differences between treatments (*P*-time × treatment = 0.629). In the present study we included male and female subjects without the intent of making a specific gender comparison. However, for general interest, muscle protein synthesis rates in the male compared with the female volunteers are presented separately in **Supplemental Table 1**.

**FIGURE 6:**
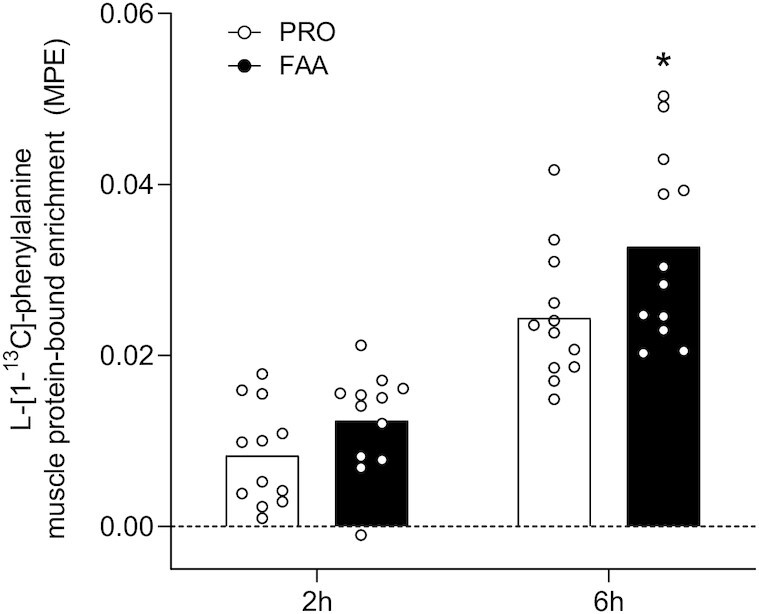
l‐[1‐^13^C]‐phenylalanine incorporation into muscle protein after ingesting 30 g PRO or an equivalent amount of FAAs. Bars are means (*n* = 12) and dots represent individual values. *Significantly different from PRO (*P* = 0.043). FAA, free amino acid; MPE, mole % excess; PRO, milk protein.

Muscle protein-bound l‐[1‐^13^C]‐phenylalanine enrichments significantly increased following FAA and PRO ingestion to 0.012 ± 0.006 compared with 0.008 ± 0.006 MPE (at *t* = 2 h) and 0.033 ± 0.011 compared with 0.024 ± 0.008 MPE (at *t* = 6 h), respectively (*P*-time < 0.001; *P*-time × treatment = 0.060; **Figure 6**). No significant differences in muscle protein-bound l‐[1‐^13^C]‐phenylalanine enrichments were observed between treatments at 2 h (*P =* 0.105). However, significantly higher muscle l‐[1‐^13^C]‐phenylalanine enrichments were observed following FAA compared with intact PRO ingestion at 6 h (*P =* 0.043).

### Muscle molecular signaling responses

The phosphorylation status (ratio of phosphorylated to total protein) of key signaling proteins involved in the initiation of muscle protein synthesis are shown in **Supplemental Figure 3**. No significant changes were observed over time for muscle mammalian target of rapamycin (mTOR) (Ser^2448^) phosphorylation status and no differences were observed between treatments (Supplemental Figure 3A). A significant time × treatment interaction was observed for muscle p70S6K (Thr^389^) phosphorylation status (*P* = 0.007) (Supplemental Figure 3B). Muscle p70S6K (Thr^389^) phosphorylation status significantly increased from 0 to 2 h (*P =* 0.003) following FAA when compared with PRO ingestion. Muscle rpS6 (Ser^235/236^) phosphorylation status (Supplemental Figure 3C) was significantly higher at the 2-h compared with the 0- and 6-h time points for both the FAA and PRO treatment (*P*-time = 0.009), with no differences between treatments (*P*-treatment = 0.591; *P*-time × treatment = 0.495). No changes and differences between treatments were observed for muscle 4E‐BP1 (Thr^37/46^) phosphorylation status over time (Supplemental Figure 3D).

## Discussion

The present study showed more rapid amino acid absorption following ingestion of FAA than with the ingestion of an equivalent amount of intact PRO in young, healthy adults. The higher rate of amino acid absorption resulted in greater postprandial phenylalanine availability, with 76% compared with 59% of the ingested amino acids being released into the circulation following ingestion of FAA compared with intact PRO. Ingestion of both intact PRO and the equivalent amount of FAA robustly increased mixed muscle protein synthesis rates, with no differences between treatments.

Previous work has shown that more rapid protein digestion and amino acid absorption leads to greater postprandial amino acid release into the circulation and greater stimulation of muscle protein synthesis rates (5, 6, 8). Consequently, it is generally assumed that ingestion of FAA as opposed to intact PRO will further augment postprandial plasma amino acid availability, resulting in a more pronounced muscle protein synthetic response (5, 8, 27). However, only a few studies have compared the postprandial release of amino acids into the circulation following the ingestion of FAA with intact PRO and/or its subsequent impact on postprandial muscle protein synthesis rates (27, 28). To our knowledge, no study has ever quantified postprandial release of exogenous amino acids and the subsequent muscle protein synthetic response following the ingestion of intact PRO compared with an equivalent amount of FAA in vivo in humans.

In the present study, we observed a greater rise in circulating amino acids following the ingestion of 30-g FAA than with the equivalent amount of intact PRO (Figures 1–2). The plasma amino acid responses (iAUC) assessed over the entire 6-h postprandial period were substantially greater following ingestion of FAA than with intact PRO. As amino acid levels did not reach baseline values with the 6-h postprandial period, it is likely that the iAUC could have increased more if the assessment period had been extended further (14). Peak plasma leucine concentrations were nearly 2-fold higher following ingestion of FAA than they were following ingestion of intact PRO (Figure 1B), despite the fact that both treatments provided exactly the same amount of leucine. The present study expands on previous work by combining continuous intravenous l-[ring-^2^H_5_]-phenylalanine infusion with the ingestion of intrinsically l-[1-^13^C]-phenylalanine–labeled PRO or an equivalent amount of FAA enriched with free l-[1-^13^C]-phenylalanine, which allows us to quantify protein digestion and amino acid absorption kinetics (14). Ingestion of FAA showed a more rapid rise in plasma l-[1-^13^C]-phenylalanine enrichments when compared with intact PRO (Figure 3A). In line with these findings, exogenous phenylalanine appearance rates were substantially higher following ingestion of FAA than with the ingestion of intact PRO during the first 2 h of the postprandial period (Figure 5A). Ingestion of amino acids consumed as free crystalline amino acids as opposed to intact PRO allowed ∼17% more of the provided phenylalanine to become available in the circulation, substantially increasing the availability of exogenous amino acids as substrates for peripheral tissues. Of course, the observed differences in amino acid absorption kinetics following PRO compared with FAA ingestion may be modulated when PRO or FAA are ingested within the matrix of a product or meal containing other (macro)nutrients and fibers (25, 29, 30).

On a whole-body level, the ingestion of FAA or intact PRO increased protein synthesis rates and lowered protein breakdown rates, resulting in a net positive protein balance (Figure 6). The greater exogenous amino acid release into the circulation following FAA compared with intact PRO ingestion resulted in a greater positive net protein balance (Figure 6), indicating greater postprandial tissue protein accretion. However, whole-body protein kinetics do not necessarily reflect skeletal muscle per se (24, 31, 32). As we also collected several skeletal muscle biopsies we were able to assess the impact of the greater postprandial amino acid release following FAA compared with intact PRO ingestion on muscle protein synthesis rates and the direct incorporation rate of dietary (protein-)derived phenylalanine into muscle protein (16, 33). In short, despite the greater release of amino acids and higher plasma leucine concentrations, no differences were observed in postprandial muscle protein synthesis between treatments.

It has been well established that the ingestion of 20 g protein is sufficient to maximize postprandial muscle protein synthesis rates in young, active individuals (34, 35). Therefore, it could be speculated that the 30-g bolus of PRO or FAA provided in the present study prevented us from detecting differences in postprandial muscle protein synthesis rates between treatments. The difference in anabolic response between treatments was lacking despite a much greater postprandial plasma amino acid release (Figure 1) and concomitant greater p70S6 phosphorylation (Supplemental Figure 3) observed following FAA compared with intact PRO ingestion. The proposed benefits of greater postprandial plasma amino acid release on stimulating muscle protein synthesis may become more evident in conditions where <20 g protein is ingested or in those populations that suffer from anabolic resistance. In the presence of anabolic resistance, ingestion of more protein is required to maximize postprandial muscle protein synthesis rates (36, 37). Ingestion of FAA as opposed to intact PRO could provide some benefits in this setting as more of the ingested amino acids will become available to the muscle, thereby compensating, at least partly, for some of the anabolic resistance. This is supported by the observation that more of the ingested l‐[1‐^13^C]‐phenylalanine was released into the circulation and used as precursor for de novo muscle protein accretion when ingested as free, crystalline phenylalanine as opposed to protein-bound phenylalanine (Figure 6). This will be even more relevant in conditions where anabolic resistance is attributed to impairments in protein digestion and/or amino acid absorption, such as in intensive care unit patients and patients suffering from gastrointestinal diseases (38, 39). Follow-up studies should focus on the proposed benefits of FAA provision in situations where protein intake is restricted and/or in older and more clinically compromised patient populations.

In conclusion, ingestion of a single bolus of FAA is followed by more rapid amino acid absorption, greater postprandial plasma amino acid release into the circulation, and greater dietary phenylalanine incorporation into mixed muscle protein than is the ingestion of an equivalent amount of intact PRO. The postprandial increase in muscle protein synthesis rate does not differ following the ingestion of 30 g intact PRO or the equivalent amount of FAA in vivo in healthy, young adults.

## Supplementary Material

nxab305_Supplemental_File

## Notes

Ajinomoto OmniChem NV provided the amino acid mixture used in this study in kind. Ajinomoto was not involved in any part of the design, analysis, or interpretation of the study.

Author disclosures: IWKK has received speaking honoraria from Nutricia Research. LJCvL and LBV have received research grants, consulting fees, and/or speaking honoraria from Friesland Campina and Nutricia Research. MCGvdP has received research grants, consulting fees, and/or speaking honoraria from Nestlé, Nutricia Research, and Fresenius-Kabi. All other authors report no conflicts of interest.

MEGW and RJJvG share first authorship.

Supplemental Methods, Supplemental Figures 1–3, and Supplemental Table 1 are available from the “Supplementary data” link in the online posting of the article and from the same link in the online table of contents at https://academic.oup.com/jn/.

Abbreviations used: EAA, essential amino acid; En%, Energy%; FAA, free amino acid; FSR, Fractional synthesis rate; GC-P-IRMS, Gas chromatography–pyrolysis-isotope ration mass spectrometer; iAUC, Incremental area under the curve; MPE, mole % excess; PRO, Milk protein; R_a_, Rate of appearance; R_d_, Rate of disappearance; TBDMS, Tert-butyldimethylsilyl.
