# Supplementary material for: Ingestion of Free Amino Acids Compared with an Equivalent Amount of Intact Protein Results in More Rapid Amino Acid Absorption and Greater Postprandial Plasma Amino Acid Availability Without Affecting Muscle Protein Synthesis Rates in Young Adults in a Double-Blind Randomized Trial
Source: J Nutr. 2021 Oct 12;152(1):59–67. doi: 10.1093/jn/nxab305 (PMC8754581; doi:10.1093/jn/nxab305)
Supplement: nxab305_Supplemental_File [file nxab305_supplemental_file.pdf]

Supplemental Figure 1

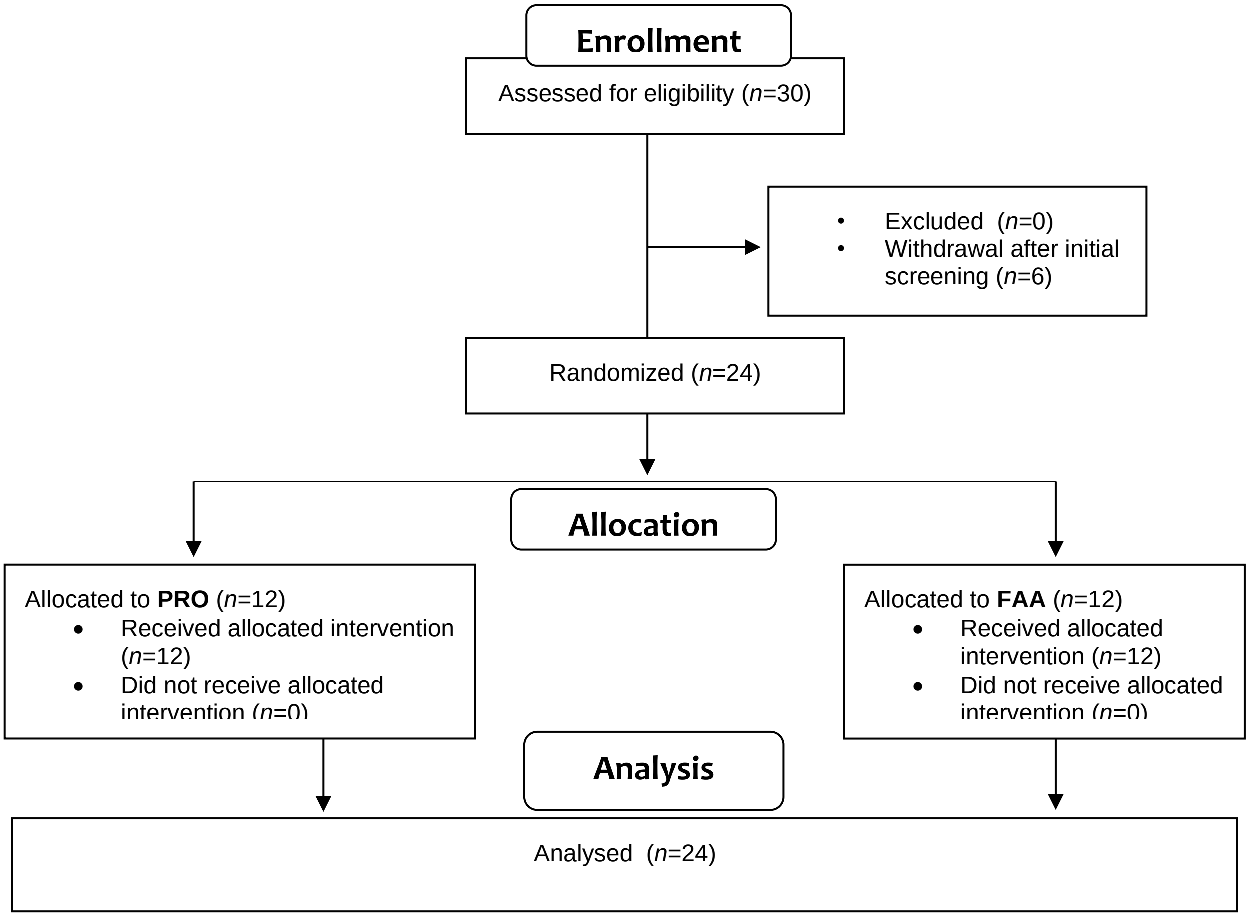

Supplemental Methods

Plasma analysis

Plasma insulin concentrations were determined with radioimmunoassay (RIA) kits (Human Insulin specific RIA, Millipore Corporation, MA, USA). Plasma amino acid concentrations were measured using ultra-performance liquid chromatograph-mass spectrometry (UPLC-MS; ACQUITY UPLC H-Class with QDa; Waters, Saint-Quentin, France). Fifty µL plasma was deproteinized using 100 µL of 10% SSA with 50 µM of MSK-A2 internal standard (Cambridge Isotope Laboratories, Massachusetts, USA). Subsequently, 50 µL of ultra-pure demineralized water was added and samples were centrifuged. After centrifugation, 10 µL of supernatant was added to 70 µL of Borate reaction buffer (Waters, Saint-Quentin, France). In addition, 20 µL

of AccQ-Tag derivatizing reagent solution (Waters, Saint-Quentin, France) was added after which the solution was heated for 10 min at 55°C. Of this 100 µL derivative 1 µL was injected and measured using UPLC-MS.

Plasma L-[ring-$^{2}H_5$]-phenylalanine and L-[ring-3,5-$^{2}H_2$]-tyrosine enrichments were determined by gas chromatography-mass spectrometry analysis (GC-MS; Agilent 7890A GC/5975C; MSD, Little Falls, USA). Specifically, internal standards of [U-$^{13}C_9{}^{15}N$]-phenylalanine and [U-$^{13}C_9{}^{15}N$]-tyrosine were added to the plasma samples. Plasma samples were deproteinized on ice with 45% 5-sulfosalicylic acid solution. Free amino acids were purified using cation exchange chromatography (AG 50W-X8 resin, 100–200 µm dry mesh size; Bio-Rad Laboratories, Hercules, USA). The purified amino acids were converted into tert-butyldimethylsilyl (TBDMS) derivatives with *N*-*tert*-butyldimethylsilyl-*N*-methyltrifluoroacetamide (MTBSTFA) before analysis by GC-MS. The amino acid concentrations were determined using electron impact ionization by monitoring ions at mass/charge (*m/z*) 336 and 346 for unlabeled and [U-$^{13}C_9{}^{15}N$] labeled-phenylalanine respectively, and 466 and 476 for unlabeled and [U-$^{13}C_9{}^{15}N$]-tyrosine, respectively. Plasma phenylalanine and tyrosine ^{13}C or ^{2}H enrichments were determined by using electron ionization and selective ion monitoring at mass/charge (m/z) 336, 337, and 341 for unlabeled and labeled (1-^{13}C and ring-$^{2}H_5$) phenylalanine, respectively, and m/z 466, 467, 468, and 470 for unlabeled and labeled (1-^{13}C, ring-$^{2}H_2$, and ring-$^{2}H_4$) tyrosine, respectively. Standard regression curves were applied from a series of known standard enrichment values against the measured values to assess the linearity of the mass spectrometer and to account for any isotope fractionation which may have occurred during the analysis.

Muscle analysis

For the measurement of mixed muscle protein-bound and muscle free L-[ring-$^{2}H_5$]-phenylalanine and L-[1-^{13}C]-phenylalanine enrichments, ~50 mg of wet muscle tissue was

freeze-dried. Collagen, blood, and other non-muscle fiber material were removed from the muscle fibers under a light microscope. The isolated muscle fiber mass (8 mg) was weighed, and 35 volumes (7 times dry weight of isolated muscle fibers wet:dry ratio) of ice-cold 2% perchloric acid were added. The tissue was then sonicated and centrifuged. After centrifugation, the supernatant was collected for determination of L-[ring-$^{2}H_5$]-phenylalanine and L-[1-^{13}C]-phenylalanine enrichments in the muscle-free amino acid pool and processed in the same manner as the plasma samples for isotopic enrichment using GC-MS analysis. The protein pellet was washed with 3 additional 1.5 mL washes of 2% PCA, hydrolysed with 6M HCl at 120°C for 15-18 h, and then dried under a nitrogen steam while heated to 120°C. Next, 50% acetic acid solution was added, and the hydrolyzed protein was passed over a cation exchange resin (Dowex AG 50W-X8, 100–200 mesh hydrogen form: Bio-Rad, Hercules, CA) using 2M NH_4OH. The eluate was dried, and the purified amino acids were derivatized to their N(O,S)-ethoxycarbonyl ethyl esters. For measurement of L-[1-^{13}C]-phenylalanine, derivatized samples were measured using a GC-C-IRMS (MAT 253; Thermo Fisher Scientific, Bremen, Germany) equipped with DB5MS 30m column (No. 122–4762; Agilent) and GC-Isolink monitoring of ion masses 44, 45. To determine the L-[ring$^{2}H_5$]-phenylalanine enrichment of mixed muscle protein, the eluate was dried, and the purified amino acids were derivatized to their N(O,S)-ethoxycarbonyl ethyl esters (23). The derivatized samples were measured using a gas chromatography-pyrolysis-isotope ration mass spectrometer (GC-P-IRMS) (MAT 253; Thermo Fisher Scientific, Bremen, Germany) equipped with a DB17MS 60m column with 5 m pre-column (No. 122–4762; Agilent) and GC-Isolink, monitoring of ion masses ^{1}H and ^{2}H. Standard regression curves were applied from a series of known standard enrichment values against the measured values to assess the linearity of the mass spectrometer and to account for any isotope fractionation which may have occurred during the analysis.

Western blotting

Western blot analysis was performed on muscle tissue (at *t*=0, 2, and 6 h). A portion of each muscle sample frozen for biochemical analyses was homogenized in seven volumes Tris buffer (20 mM Tris-HCL, 5 mM EDTA, 10 mM Na pyrosphospate, 100 mM NaF, 2 mM Na_3VO_4, 1% Nonident P-40; pH 7.4) supplemented with protease and phosphatase inhibitors: aprotinin 10 µg/mL, leupeptin 10 µg/mL, benzamidin 3 mM and phenylmethylsulphonyl fluoride 1 mM. After homogenization, each muscle extract was centrifuged for 10 min at 10 000 *g* (4 °C) and sample buffer was added to the supernatant to final concentrations of 60 mM Tris, 10% glycerol, 20 mg/mL SDS, 0.1 mM dithiothreitol, and 20 µg/mL bromophenol blue. The supernatant was then heated for 5 min at 100°C and immediately placed on ice. Immediately before analyses, the muscle extraction sample was warmed to 50°C and centrifuged for 1 min at 1000*g* at room temperature. The total amount of sample loaded on the gel was based on protein content. After a Bradford assay, 30 µg protein were loaded in each lane. With the exception of mTOR, protein samples were run on a Criterion Precast TGX 4–20% gel (Order No. 567–1094; Bio-Rad) ± 90 min at 150 V (constant voltage) and transferred onto a Trans-blot Turbo 0.2 µm nitrocellulose membrane (Order No. 170–4159; Bio-Rad) in 7 min at 2.5 A and 25 V. The mTOR proteins were run and blotted for 10 min at 2.5 A and 25 V but on a Criterion Precast XT 3–8% Tris-acetate gel (Order No. 345-0130; Bio-Rad). Specific proteins were detected by overnight incubation at 4°C on a shaker with specific antibodies in 50% PBS Odyssey blocking buffer (Part No. 927-40 000; Li-Cor Biosciences, Lincoln, NE, USA) after blocking for 60 min at RT in 50% in PBS Odyssey blocking buffer. Polyclonal primary phospho-specific antibodies, anti-phospho-mTOR (Ser^{2448}), anti-phospho-S6K1 (Thr^{389}), anti-phospho-rpS6 (Ser^{235}/Ser^{236}) and anti-phospho-4E-BP1 ($Thr^{37/46}$) were purchased from Cell Signaling Technology (Danvers, MA, USA). In addition, anti-mTOR, anti-S6K1, anti-RS6 and anti-4E-BP1 were also purchased from Cell Signaling Technology. Following incubation, membranes were washed three times

10 min in 0.1% PBS-Tween 20 and once for 10 min in PBS. Next, samples were incubated on a shaker (1 h at RT) with infrared secondary antibodies, donkey anti-rabbit IRDYE 800 (dilution 1:10 000; Cat. No. 611-732-127; Rockland Immunochemicals, Pottstown, PA, USA) and donkey anti-mouse IRDYE 800CW (dilution 1:10 000; Cat. No. 626–32 212; Li-Cor Biosciences) dissolved in 50% PBS Odyssey blocking buffer. After a final wash step (3 × 10 min) in 0.1% Tween 20-PBS and once 10 min in PBS, protein quantification was performed by scanning on an Odyssey Infrared Imaging System (Li-Cor Biosciences). Ponceau S staining was used to standardize for the amount of protein loaded. Phosphorylation status as a proxy of activation of the signaling proteins was expressed relative to the total amount of each protein.

Supplemental Table 1: MPS data for males and females

	Males		Females	
	PRO (*n*=6)	FAA (*n*=6)	PRO (*n*=6)	FAA (*n*=6)
Basal (%·h^{-1})	0.030±0.002		0.046±0.005	
0-2h (%·h^{-1})	0.049±0.012	0.060±0.005	0.072±0.031	0.067±0.031
2-6h (%·h^{-1})	0.048±0.020	0.043±0.009	0.046±0.015	0.056±0.020

FAA, free amino acids; PRO, milk protein;

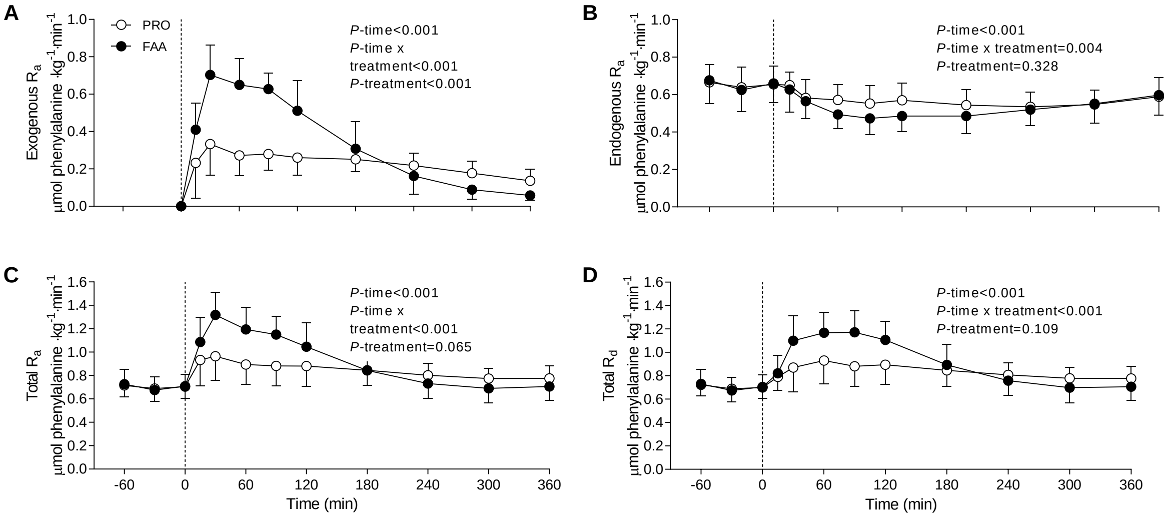

Supplemental figure 2: Exogenous rate of appearance (R_a; **A**), endogenous R_a (**B**), Total R_a (**C**), and Total rate of disappearance (R_d; **D**) after ingesting 30 g milk protein (PRO; *n*=12) or an equivalent amount of free amino acids (FAA; *n*=12). The dashed line refers to drink ingestion. Data were analyzed with repeated measures ANOVA. FAA, free amino acids; PRO, milk protein.

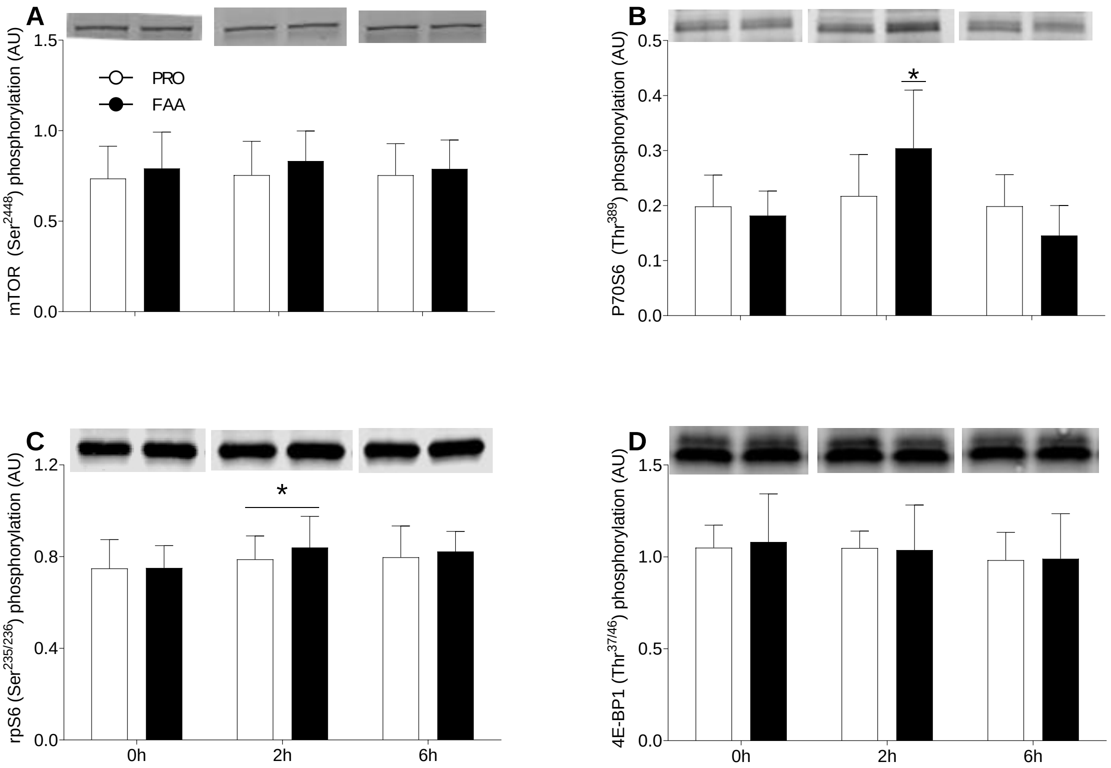

Supplemental figure 3: Skeletal muscle phosphorylation status (ratio of phosphorylated to total protein) of mTOR (Ser^{2448}) (**A**), p70S6K (Thr^{389}) (**B**), rpS6 ($Ser^{235/236}$) (**C**) and 4E-BP1 ($Thr^{37/46}$) (**D**) in the basal state, (*t*=0 h) and at 2 and 6 h after ingesting 30 g milk protein (PRO; *n*=12) or an equivalent amount of free amino acids (FAA; *n*=12). *Significantly different from *t*=0 h (*P*<0.05). FAA, free amino acids; 4E-BP1, eukaryotic translation initiation factor 4E-binding protein-1; mTOR, mammalian target of rapamycin; p70S6K, p70S6 protein kinase 1; PRO, milk protein; rpS6, ribosomal protein S6.
